# Spontaneous duodenal perforation in a neonate: A case report

**DOI:** 10.1016/j.radcr.2025.12.052

**Published:** 2026-01-30

**Authors:** Zelalem Assefa Semegn, Chibahew Lante Kebede

**Affiliations:** Pediatric Surgery Unit, Surgery Departement, Saint Paul Hospital Millennium Medical College, Addisabeba, Ethiopia

**Keywords:** Neonatal duodenal perforation, Spontaneous perforation, Pneumoperitoneum, Abdominal radiography, Football sign, Rigler’s sign, Neonatal surgery

## Abstract

Neonatal duodenal perforation is an extremely rare but life-threatening surgical emergency. Reported etiologies include prematurity, necrotizing enterocolitis, distal obstruction, trauma, and sepsis; however, in rare instances, no identifiable cause is found and the condition is described as *spontaneous*. Early diagnosis relies heavily on *radiologic imaging*, particularly plain abdominal radiographs demonstrating *pneumoperitoneum*. We report a 2-day-old full-term female neonate who presented with progressive abdominal distension, nonbilious vomiting, and low-grade fever of one day duration. Laboratory evaluation showed leukocytosis with a high absolute neutrophil count and mildly elevated C-reactive protein; blood culture grew *Klebsiella pneumoniae*, for which appropriate antibiotic therapy was initiated. *Supine abdominal radiography revealed massive pneumoperitoneum with classic radiologic signs*, including the *football sign and Rigler’s (double-wall) sign*, consistent with gastrointestinal perforation. Exploratory laparotomy identified a solitary perforation on the anterior wall of the first part of the duodenum, with no evidence of distal obstruction, necrotizing enterocolitis, ischemia, or traumatic injury. The perforation was repaired by primary closure reinforced with a pedicled omental patch. Despite the presence of bacteremia, no intraoperative findings supported sepsis-related bowel necrosis, and the perforation was therefore classified as spontaneous. The neonate had an uneventful postoperative recovery. Although exceedingly rare, neonatal duodenal perforation carries significant morbidity and mortality. *Prompt radiologic recognition of pneumoperitoneum and early surgical intervention* are critical for favorable outcomes. This case highlights the diagnostic value of imaging and underscores the importance of carefully excluding infectious and secondary causes before labeling a duodenal perforation as spontaneous.

## Introduction

Duodenal perforation in neonates is uncommon condition [[1], [2], [3]]. Most of duodenal perforations are located on the anterior duodenum [3]. Features that suggest peritonitis or pneumoperitoneum include distended and tender abdomen, respiratory distress, thrombocytopenia, metabolic acidosis, failure to pass feces, and vomiting [1,4,5]. Duodenal perforation in a neonate is usually managed by primary repair [1,2,6,7].

We are reporting a 2 days old, female neonate diagnosed with a spontaneous perforation at the first part of the duodenum anteriorly.

## Case presentation

A full term, female neonate with a birth weight of 2900 gram was born from 32 years old, para III mother by vaginal delivery. APGAR scores were 7 and 8 in the 1st and 5th minute. Labor was induced for the indication of prolonged rupture of membrane. The mother had regular ANC follow up, and it was uneventful. The mother had no history of use of medications like steroids and NSAIDs.

The neonate presented with abdominal distension, vomiting of ingested matter, and low-grade fever of 1-day duration. On examination, she had fever of 38.1°C, distended and tender abdomen. Laboratory investigations showed ANC of 28,464/mL, platelet of 110, 000/mL, and CRP of 3-10 mg/L. Blood culture revealed growth of *K. pneumoniae* postoperatively on 4th post op day. An abdominal x ray showed significant pneumoperitoneum (Image 1).

With the above findings, the neonate was diagnosed with generalized peritonitis secondary to perforated viscus. Then, she was resuscitated and operated. Laparotomy revealed around 150 mL upper GI content in the general peritoneum and 0.5cm by 0.5cm perforation on the anterior aspect of the 1st part of duodenum. There was no distal obstruction or mass. The rest of the bowel was examined and it appeared normal. Primary closure of the perforation was done in single layer. An onlay pedicled omental patch was applied. Nasojejunal and orogastric tubes were placed. A drain was also placed around the repair site. Since the visualized mucosa around the perforation was normal, a biopsy was not taken to avoid the risk of future stricture formation.

**Fig. 1:**
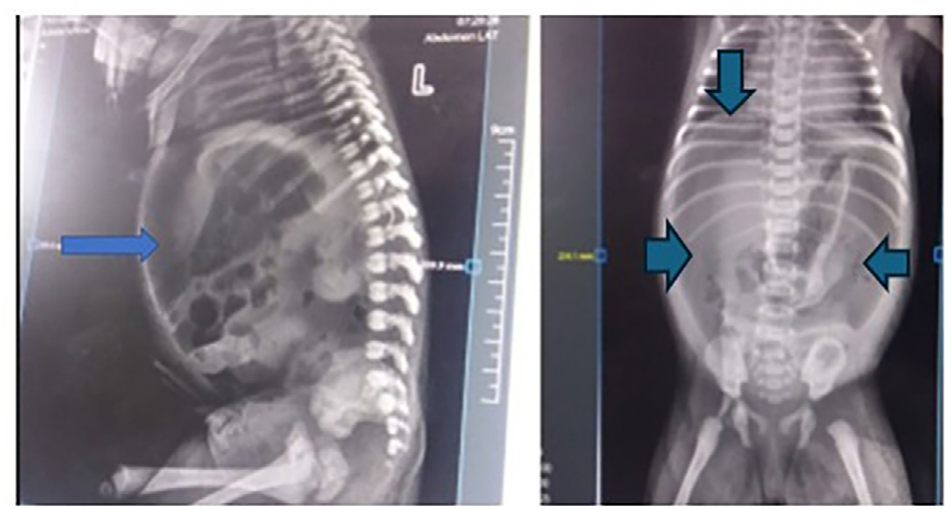
AP and lateral x-ray showing significant pneumoperitoneum.

On the 3rd postoperative day, feeding was started thru the Nasojejunal tube. Feeding was escalated, and full feeding was attained on the 6th postoperative day. Drain and Nasojejunal tubes were removed on the 7th postoperative day. The neonate recovered uneventfully. She was discharged on the 16th postoperative day after completion of antibiotics. On follow up, she was found to be healthy and thriving well Figure 1.

## Discussion

Gastrointestinal perforation in a newborn is rare [2,4,8]. Perforation has been reported to occur in the stomach, the duodenum, and the small and large bowel [8]. Gastric and duodenal perforation occur less frequently than perforation of the rest of bowel [8,9]. Perforation associated with risk factors is relatively common [4,[8], [9], [10]]. Although rare, gastrointestinal tract perforation can occur in neonates spontaneously in the absence of factors known to cause perforations like peptic ulcer disease, asphyxia, sepsis, trauma from intubation, and distal obstruction [1,2,4,[8], [9], [10]].

Duodenal perforation in a neonate is uncommon, with the majority occurring in the anterior duodenum [[1], [2], [3],^6^]. The following risk factors has been mentioned on literatures: peptic ulcer disease, use of ventilators, prolonged use of oxygen via nasal cannula or face mask, sepsis, enteral tube feedings, high acid secretions in newborns in the 1st 10 days due to high maternal gastrin, maternal use of steroids and NSAIDs, prematurity, and low birth weight [[1], [2], [3],^5^,^10^,^11^]. In some of the cases, a cause could not be found and these are reported as ``spontaneous'' to describe a perforation without a known underlying cause [1,2]. However, most suggest when a gastrointestinal perforation occurs in a neonate, an underlying cause should be looked for.

Duodenal perforations are associated with high mortality, especially if diagnosis and intervention is delayed [1]. Therefore, early identification and management is prudent to improve patient outcome. Its management is primary closure with or without omental patch [2,3,7].

In our case, the neonate had no risk factor. She was a healthy, full term, normal birth weight neonate. And she was not on enteral tube feeding, oxygen or mechanical ventilator. To the best of our knowledge, a spontaneous duodenal perforation has been reported twice before.

## Conclusion

Duodenal perforations are associated with high mortality [1]. Therefore, physicians should know the causes of duodenal perforation. This allows earlier identification of perforation, and improvement of patient outcome. Most of duodenal perforations can be managed by primary closure and this should be considered as the management of choice [1,2,[5], [6], [7]].

Lateral view of the abdomen showing free intraperitoneal air outlining the anterior abdominal wall pointed by large arrows. The lucent area beneath the diaphragm is consistent with pneumoperitoneum.

## Anteroposterior abdominal X-ray showing the “football sign”

Frontal abdominal radiograph demonstrating the classic “football sign,” with free intraperitoneal air outlining the peritoneal cavity and falciform ligament shown by small wide arrow heads. The oval, radiolucent appearance resembles an American football, indicating massive pneumoperitoneum.

## Ethics approval

According to the review board policy of our institution, ethics approval was not necessary. Written consent was obtained from the patient’s family for publication of this case report and associated images.

## Declaration of generative AI and AI-assisted technologies in the manuscript preparation process

During the preparation of this work the author(s) used chatGpt in order to improve language and readability. After using this tool/service, the author(s) reviewed and edited the content as needed and take(s) full responsibility for the content of the published article.

## Ethical approval

Ethical approval is deemed unnecessary by the hospital ethics committee as this is a rare case faced during clinical practice and doesn't involve experiments in humans or animals.

## Patient consent

Written informed consent was obtained from the patient for publication and use of images. The written consent is available for review by the Editor-in-Chief of this journal upon inquiry
